# Preconception care utilization and pregnancy outcomes among postpartum women at Komfo Anokye Teaching Hospital, Ghana

**DOI:** 10.3389/frph.2025.1509737

**Published:** 2025-04-03

**Authors:** Timothy Kwabena Adjei, Opei Kwafo Adarkwa, Evans Ansu-Yeboah, Esmond Ofori, Bernard Arhin, Augustine Tawiah, Charles Mawunyo Senaya, Seth Amponsah Tabi, Amponsah Peprah, Edward Tieru Dassah, Atta Owusu Bempah

**Affiliations:** ^1^ Department of Obstetrics and Gynecology, School of Medicine and Dentistry, College of Health Sciences, Kwame Nkrumah University of Science Technology, Kumasi, Ghana; ^2^ Directorate of Obstetrics and Gynecology, Komfo Anokye Teaching Hospital, Kumasi, Ghana; ^3^ Research and Development Unit, Komfo Anokye Teaching Hospital, Kumasi, Ghana

**Keywords:** preconception care, utilization, postpartum women, maternal outcomes, adverse pregnancy outcomes, Ghana

## Abstract

**Introduction:**

Maternal and perinatal morbidities are alarming in Sub-Saharan Africa. However, most of these can be prevented through appropriate care and interventions including preconception care (PCC). There is paucity of data on the effect of PCC on pregnancy outcomes in Ghana. This study sought to determine the association between PCC utilization and late pregnancy outcomes among postpartum women at Komfo Anokye Teaching Hospital (KATH). The study also assessed factors associated with its utilization.

**Method:**

A total of 336 postpartum women from an unmatched 1:2 case-control study, were interviewed. Women with late adverse pregnancy outcomes (APO) in the index pregnancy constituted the case group while those with no APO made up the control group. For every case who gave consent, two consecutive controls were recruited until the sample size was attained. Categorical variables were compared using Chi-square (*χ*2) or Fisher's exact test as appropriate, while continuous variables were compared using student t-tests. Multivariable logistic regression analysis was performed to estimate the odds ratios and the association between PCC utilization and pregnancy outcomes as well as factors associated with PCC utilization.

**Results:**

A total of 112 cases and 224 controls were analyzed with comparable mean ages (Cases-30.2 ± 5.97 vrs Controls-30.5 ± 5.89 years, *p* = 0.45). PCC utilization rates were significantly lower among women who suffered late APO (14.3%) than those who did not (25.0%) *p* = 0.0241. PCC utilization was protective of late APO (OR-0.582) but not statistically significant (95% CI, 0.256–1.324; *p* = 0.197). Factors associated with PCC uptake included pregnancy intention (OR- 22.781; 95% CI, 7.883–65.837; *p* = 0.001), knowledge of PCC (OR- 56.4; 95% CI, 16.105–197.517; *p* = 0.001) and pre-existing medical condition (OR-3.976; 95% CI, 1.009–15.677, *p* = 0.049).

**Conclusion:**

PCC utilization rates are low among postpartum women. Women who utilized PCC were twice less likely to suffer any late APO outcome compared to those who did not, though this was not statistically significant. Knowledge of PCC, pregnancy intention, and the presence of pre-existing medical conditions are factors associated with PCC utilization. These findings underscore the need for enhanced PCC education and targeted interventions to improve its utilization, particularly among women at high risk of APO.

## Background

Globally, research has established that every minute, 380 women become pregnant and 190 face unplanned or unwanted pregnancies; 110 experience pregnancy-related complications, and one woman dies from a pregnancy-related cause (1). Maternal mortality continues to be a huge burden globally with over 94% occurring in Low- and middle-income countries including Ghana (LMICs) (2). Adverse pregnancy outcomes undoubtedly have been found to have serious health consequences for the mother and the offspring (3). In Ghana, although much efforts have been targeted in improving antenatal care services leading to an astronomical increase of 98% in coverage (4), maternal and perinatal morbidities and mortalities have not significantly declined over the past decades: maternal mortality rate of 484 per 100,000 live births in 2,000 to 308 per 100,000 live births in 2017 (5). These figures highlight the urgent need for comprehensive interventions throughout the reproductive continuum, including preconception care, to improve health outcomes for women and children in Ghana.

Preconception care is one of the proven strategies for the reduction in mortality and adverse health effects for the woman, fetus, and newborn by optimizing maternal health services and improving maternal health outcomes (6, 7). According to the World Health Organization (WHO), preconception care (PCC) is the provision of biomedical, behavioral, and social health interventions to women and couples before conception occurs, to improve their health status, and mitigate behaviors, individual and environmental factors that could contribute to poor maternal and child health outcomes (1, 8). The integration of PCC into primary health care to enhance utilization for all women has been touted by several bodies including WHO (7). The primary health care level should be able to offer the basic package of PCC such as folic acid supplementation while the extended package may be limited to secondary or tertiary levels of care (1).

Despite the proven clinical value and cost-effectiveness of PCC, there is low uptake of the service especially in LMICs where it's most needed (3) compared to that of developed countries where utilization rates range from 35% to 75% (9). The prevalence of utilization of PCC in Africa (18.72%) and Ghana (15%) is remarkably low (6). Strategies for implementation of PCC will require clinical, public health, consumer, research and surveillance, policy, and financial considerations (10).

There is no specific policy of PCC in Ghana presently and any policy direction to improve access to PCC services and integrate it into our health system will be grounded on the quality of evidence on PCC utilization and its effects in our settings. However, previous studies in Ghana on PCC just looked at its awareness, knowledge and prevalence but did not determine the effect of PCC on pregnancy outcomes (11, 12). The effect of PCC practices on pregnancy outcomes among women in our setting is unknown as well as factors associated with PCC utilization, especially among mothers in urban areas where this service could be accessed. This study sought to determine the association between PCC utilization and late pregnancy outcomes among postpartum women at the Komfo Anokye Teaching Hospital. The study also identified the factors associated PCC utilization.

## Methodology

### Study design and settings

The study employed a case-control design among post-partum women. Although it has the potential limitation of recall bias, this design was adopted because it is the most feasible and cost-effective design to address the research questions within the time frame. The study was conducted in the obstetric unit of Komfo Anokye Teaching Hospital, Kumasi; the second leading hospital in Ghana. This hospital is a referral centre for most health facilities from the middle half to the northern regions of the country. The Department of Obstetrics and Gynaecology has a bed capacity of 204 and undertakes about 6,000 deliveries a year with an average monthly delivery of 500. The hospital recorded a maternal mortality ratio of 1,818.18 per 100,000 live births, 339 stillbirths, and a caesarean section rate of 50.47% in the year 2021. Komfo Anokye Teaching Hospital presently offers the basic package and some forms of the expanded package of PCC services. These services in the department are limited usually at the family planning unit and antenatal care clinic.

### Study population

This study was conducted among women who delivered at the Komfo Anokye Teaching Hospital. Women with at least, one late adverse pregnancy outcome in the index pregnancy who consented to the study constituted the case group while those with no late adverse pregnancy outcomes made up the control group. For our study, pregnancy with late adverse outcome was defined as pregnancy that resulted in maternal and/or feto-neonatal compromise occurring at Estimated Gestational Age (EGA) of ≥28 weeks. Inclusion criteria for the study comprised, women who had outcomes of their pregnancies occurring at ≥28 weeks of gestation at KATH, women within the puerpera, and are at least 18 years of age. Women who were unconscious or in a state unable to give consent were excluded from the study.

Maternal utilization of PCC service was considered the main exposure variable, For the index pregnancy, the classification of maternal utilization of PCC service was based on the mothers’ responses to 4 questions: At any time during the period from 3 months before this pregnancy, did you seek preconception care services? Please indicate the component/form of the PCC service you received. Where did you seek such a service? Who provided this care? The answers to these questions with consideration of the definition of preconception care and its utilization as stated by WHO were used to group mothers into two mutually exclusive categories: Utilizers and Non-utilizers of PCC.

### Sampling size estimation

Sample size calculations were done using OpenEpi software, version 3.01 (Open source Epidemiologic Statistics for Public Health), and a minimum total sample size of 333, with continuity correction comprising 111 cases and 222 controls was arrived at as required for unmatched case-control studies with the following parameters considered: confidence level of 95%, study power of 80%, ratio of control to cases of 2, percentage of controls exposed (prevalence of PCC in the general population) of 15% (8, 10) and an assumed odds ratio (OR) of 2.3.

In all, a total of 336 participants were recruited into the study; 112 cases and 224 controls at a ratio of 1:2 respectively. Women with at least, one late APO admitted to the ward were contacted for inclusion (including a review of their clinical records). Participants were selected by convenient sampling. For every case recruited into the study, the next two consecutive controls were contacted for inclusion. As such cases and controls were recruited consecutively until the total sample size was attained.

### Data collection, processing and analysis

Participants who satisfied the inclusion criteria were approached whiles on admission for recruitment into the study after reviewing their clinical notes on the electronic medical record system as well as their maternal health record books. Consent was sought from eligible participants. Participants who gave consent were made to sign or thumbprint the consent form. Study participants were interviewed in Twi (dominant) or English language with translation by trained research assistants. Mothers were interviewed at their convenience as early as 24 h after delivery till 6 weeks post-delivery whiles on admission. Interviewing of each study participant was within a period of not more than 15 min. Three participants were contacted for inclusion but declined, Pretested structured questionnaire was used to capture data from study participants from the 15th of March 2023 to the 31st of May, 2023. Sections within the questionnaire included information on socio-demographic background, obstetric (reproductive) profile, preconception care utilization, and factors associated with the uptake of PCC.

A database was created using ODK version 2022 for data entry. Data was then exported from ONA platform to Excel spreadsheet (Microsoft Corporation, Redmond, WA) for cleaning. Statistical tests were done with STATA 17 software (Stata Corporation, Texas, USA). Level of knowledge of PCC were scored and categorized into “low”, “average”, or “good” based on the accrued score obtained by the study participant. Preconception care was categorized into utilization of PCC and non-utilization of PCC. Descriptive statistics was done with means and standard deviations estimated for continuous variables such as age. Categorical variables were analyzed using frequencies and percentages. Categorical variables were compared using Pearson's Chi-square (*χ*^2^) or Fisher's exact test as appropriate, while continuous variables were compared using student t-tests and *p*-value of less than or equal to 0.05 was deemed statistically significant. Multivariable logistic regression analysis was performed to estimate the odds ratios and the association between preconception care utilization and pregnancy outcomes as well as predictors of PCC utilization while controlling for potential confounding factors after crude analysis. For the purpose of this study, a 95% confidence interval was used and a *p*-value < 0.05 was considered statistically significant.

### Ethical consideration

Ethical approval for this study was granted by the Komfo Anokye Teaching Hospital Institutional Review Board (KATH-IRB) with registration number, KATH IRB/AP/004/23, before conducting the study.

## Results

The mean ages among cases and controls were 30.2 ± 5.97 and 30.5 ± 5.89 years respectively (*p* = 0.45). The demographic profile of the study participants is illustrated in Table 1. Akan was the main ethnic group among study participants in both arms (cases, 67.0%, and controls, 74.1%) depicting the dominant ethnic group in the Ashanti region of Ghana. Most of the study participants were married or living together with their partners. The reproductive characteristics among cases and controls are compared in Table 2, as well as preconception care utilization among participants. Cumulatively, the preconception care utilization rate was 21.4% (cases-14.3% vs. controls-25.0%, Odd's Ratio 0.5, *p* = 0.0241, 95% CI, 0.25–0.95). Women who utilized PCC were twice less likely to develop late APOs compared to those who did not. However, when adjusted, the risk though still protective, was not statistically significant (OR-0.582, 95% CI, 0.256–1.324; *p* = 0.197) as illustrated in Table 3. The adjusted analysis of the relationship between utilization of preconception care services and the underlying factors is summarized in Table 4. After adjusting for all confounding variables in the analysis, pregnancy intention (OR-22.781; 95% CI, 7.883–65.837; *p* = 0.001), level of knowledge of PCC (OR-56.4; 95% CI, 16.105–197.517; *p* = 0.001) and pre-existing medical condition (OR-3.976; 95% CI, 1.009–15.677, *p* = 0.049) had statistically significant relationship with PCC utilization among postpartum women.

**Table 1:** Comparism of socio-demographic profiles among women with late adverse pregnancy outcomes (cases) and those without (controls).

Demographic	Case *n*-112 *n* (%)	Control *n*-224 *n* (%)	Total *N*-336	*P* value
Age category (years)				0.4517
18–20	9 (8.0)	9 (4.0)	18	
21–25	13 (11.6)	40 (17.9)	53	
26–30	40 (35.7)	70 (31.2)	110	
31–35	27 (24.1)	58 (25.9)	85	
36–40	17 (15.2)	36 (16.1)	53	
>40	6 (5.4)	11 (4.9)	17	
Mean (standard deviation)	30.2 (5.97)	30.5 (5.89)		
Educational background				0.0070
No formal education	3 (2.7)	14 (6.3)	17	
Primary	10 (8.9)	8 (3.6)	18	
Middle/Junior High School	50 (44.6)	69 (30.8)	119	
Secondary/Vocational	33 (29.5)	61 (27.2)	94	
Tertiary	16 (14.3)	72 (32.1)	88	
Employment status				0.0186
Apprentice/Student	11 (9.8)	17 (7.6)	28	
Public Servant	13 (11.6)	59 (26.3)	72	
Self Employed	79 (70.6)	136 (60.7)	215	
Unemployed	9 (8.0)	12 (5.4)	21	
Ethnic group				0.2555
Hausa	4 (3.6)	4 (1.8)	8	
Kusaasi	6 (5.4)	10 (4.5)	16	
Akan	75 (67.0)	166 (74.1)	241	
Ewe	2 (1.8)	10 (4.4)	12	
Others	25 (22.2)	34 (15.2)	59	
Marital status				0.1575
Divorced/Separated	1 (0.9)	0 (0.0)	1	
Married/Living together	99 (88.4)	209 (93.3)	308	
Single	12 (10.7)	15 (6.7)	27	
Religion				0.1163
Christian	87 (77.7)	192 (85.7)	279	
Muslim	25 (22.3)	31 (13.8)	56	
Pagan	0 (0.0)	1 (0.5)	1	

**Table 2:** Comparism of reproductive characteristics and PCC utilization among cases and controls.

Reproductive Profile	Cases *n*-112 *n* (%)	Control *n*-224 *n* (%)	Total *N*-336	*P* value
Pregnancy intention				0.4404
Yes	59 (52.7)	108 (48.2)	167	
No	53 (47.3)	116 (51.8)	169	
Parity				0.3954
1	37 (33.0)	59 (26.4)	96	
2–4	58 (51.8)	132 (58.9)	190	
≥5	17 (15.2)	33 (14.7)	50	
History of adverse pregnancy outcome				0.1851
No prior adverse outcome	62 (55.4)	133 (59.4)	195	
Prior adverse outcome	50 (44.6)	91 (40.6)	141	
Gestational age at booking				0.7204
0–12wks	49 (43.8)	99 (44.2)	148	
13–25wks	57 (50.9)	108 (48.2)	165	
>25wks	6 (5.3)	17 (7.6)	23	
Number of ANC attendance				0.0001
0	3 (2.7)	2 (0.9)	5	
1–4	23 (20.5)	30 (13.4)	53	
5–9	71 (63.4)	103 (46.0)	174	
>9	15 (13.4)	89 (39.7)	104	
Presence of pre-existing medical condition				0.4367
Yes	13 (11.6)	20 (8.9)	33	
No	99 (88.4)	204 (91.1)	303	
Knowledge level of PCC				0.0019
Low	14 (12.5)	14 (6.3)	28	
Average	72 (64.3)	117 (52.2)	189	
Good	26 (23.2)	93 (41.5)	119	
PCC utilization				0.0241
PCC utilizers	16 (14.3)	56 (25.0)	72	
PCC non-utilizers	96 (85.7)	168 (75.0)	264	

ANC, antenatal care; PCC, preconception care.

**Table 3:** Multivariate regression table of association between PCC utilization and late adverse pregnancy outcome (APO).

Late adverse pregnancy outcome	Odd's ratio	*P* value	95% Confidence interval
PCC utilization	0.582	0.197	0.256–1.324
Age	1.032	0.250	0.978–1.090
Prior adverse pregnancy outcome	1.395	0.210	0.837–2.323
Educational level	0.890	0.428	0.666–1.188
Knowledge level of PCC	0.607	0.078	0.348–1.057
Employment status	0.785	0.197	0.544–1.134
Pregnancy intention	1.527	0.106	0.9145–2.551
Parity	0.560	0.044	0.364–0.986
Pre-existing medical condition	1.373	0.429	0.626–3.009

PCC, preconception care.

**Table 4:** Multivariate regression table of factors associated with of preconception care utilization.

Factors of PCC utilization	Odd's ratio (OR)	95% confidence interval	*P* value
Prior adverse pregnancy outcome	2.148	0.811–5.689	0.124
Educational level	0.852	0.458–1.583	0.612
Level of knowledge of PCC	56.400	16.105–197.517	0.001
Employment status	1.925	0.893–4.146	0.095
Pregnancy intention	22.781	7.883–65.837	0.001
Pre-existing medical condition	3.976	1.009–15.677	0.049
Age	1.090	0.974–1.220	0.135
Parity	0.516	0.207–1.285	0.155

PCC, preconception care.

## Discussion

The study demonstrated overall preconception utilization rate was low (21.4%) among postpartum women delivering in a teaching hospital in Ghana with women who suffered late APO being twice less likely to have utilized PCC compared with those who did not. This rate is slightly higher than that of the average sub-Saharan PCC utilization rates (6) as well as other studies in northern (11) and southern Ghana (12). Research has revealed diverse disparities in PCC utilization rates across developed countries such as the United States of America (35%), China (40%), and, developing countries; Ethiopia (13%) and Kenya (25%) (9). Studies in Nigeria have identified varying utilization rates ranging from 10% in south-eastern Nigeria (13) to as high as 34.2% in Lagos (14). It is therefore not surprising to find such disparities in PCC utilization rates within various areas in the same country. The justification for this low uptake may be the absence of a well-structured PCC unit to offer comprehensive PCC services within and around the study settings. The existence of a well-structured unit for PCC delivery is a key determinant for PCC utilization (15).

It was established that women who utilized preconception care were about twice less likely to suffer any late adverse pregnancy outcome compared to those who did not although this association was not statistically significant. Statistical insignificance may not preclude clinical significance always. The importance of preconception care significantly reducing adverse pregnancy outcome cannot be overemphasized (16). The findings from our study could possibly be due to the lesser numbers of PCC utilizers studied, the study design employed, as well as residual confounding variables. Another likely reason for this outcome could be due to the study focusing on only late adverse pregnancy outcomes and hence excluding those occurring less than 28 weeks gestational age.

This study demonstrated that knowledge of preconception care was significantly associated with its utilization. Women with good knowledge of preconception care were about 56 times more likely to utilize PCC services compared with those who were less knowledgeable. This finding is consistent with what was reported in Africa (6). The level of knowledge of preconception care has been reported in Ethiopia and other numerous research works as a determinant for PCC uptake (15, 16). Attempts to increase PCC awareness will eventually enhance its uptake and consequently reduce the burden of adverse pregnancy outcomes (17). It has been reported that PCC campaign messages that focus on healthy behaviours, risk reduction, and the benefits of preconception care are likely to achieve the desired impact (8). Healthcare personnel are the main source in providing education on PCC. Effective multi-disciplinary approaches among varying disciplines of health care providers can promote the knowledge and awareness of PCC (8).

Findings from this study also established that pregnancy intention was significantly associated with preconception care utilization. Women who plan their pregnancies were about 23 times more likely to utilize preconception care services compared with those who fail to plan. This outcome was corroborated by the recent meta-analysis of PCC utilization among women in Africa (6). A study in South Africa, indicated that poor pregnancy planning negatively influenced preconception care utilization (18). Findings from this study on pregnancy planning largely corroborated with global trends as well as local surveys. About 40% of all pregnancies globally are unplanned and hence are at high risk for adverse outcomes such as abortion (2, 19). Attempts to harness this window of opportunity to empower women and couples in their reproductive years will significantly enhance the reduction of adverse pregnancy outcomes. The involvement of male partners in the processes of PCC practices will be helpful in facilitating the decision making on reproductive health.

In addition, this study discovered that the presence of pre-existing medical conditions had a strong association with uptake of preconception care. A woman with a pre-existing medical condition was 4 times more likely to utilize preconception care compared to those with no such condition. This outcome has also been established in studies in Africa (6, 20). The presence of chronic medical conditions increases a woman's risk of developing adverse pregnancy outcomes (16). Such women probably are likely to have appreciable health-seeking behaviours which make them candidates to possibly receive PCC services from their primary healthcare providers. Providers of primary health care at all levels of care who may be providing care to women with chronic medical conditions are therefore well positioned to initiate preconception care. Involving all healthcare providers is essential to the delivery of preconception care services (1). The evidence of integrating PCC into the normal health care system, especially in primary care settings, as an avenue to increase uptake has already been established (16).

## Conclusion

Preconception care utilization rate is low, especially among women who suffered adverse pregnancy outcomes compared with those who did not, in a teaching hospital in Ghana. The study established that preconception care is protective of late adverse pregnancy outcome though this association was not statistically significant. Key factors influencing PCC utilization included pregnancy intention, knowledge of PCC and the presence of pre-existing medical conditions.

## Recommendations and future research

These findings underscore the need for enhanced education on PCC and targeted interventions to improve its utilization, particularly among women who are at high risk for adverse pregnancy outcomes. Establishing a comprehensive PCC service unit within the Maternal Neonatal Child Health (MNCH) service delivery framework in the study setting and similar areas is highly recommended to improve uptake of PCC services. The findings from this study provide critical insights for policy makers and healthcare providers aiming to reduce maternal and perinatal morbidities in Ghana and other sub-Saharan countries. Data generated from this work can be used to undertake several large-scale future research works in similar settings to assess the strength and accuracy of associations between PCC and pregnancy outcomes.

## Strengths and limitations

The research work is the first to compare PCC utilization with pregnancy outcomes as well as identify factors associated with its uptake among postpartum women in a teaching hospital in Ghana. The results of this study conducted in a tertiary referral centre cannot be extrapolated or entirely generalized to other facilities, particularly lower-level facilities nationwide. Komfo Anokye Teaching Hospital receives referrals from almost seven regions in the country, unlike other hospitals. Most of these referred cases might have developed complications and hence are at high risk for adverse pregnancy outcomes. The bulk of healthcare facilities, especially those at lower levels may not experience situations like this. As with case-control studies, there could be recall bias, since participants had to recollect their exposure to the utilization of preconception care. The study relied on the accuracy of the participants' recall of preconception care and the availability and quality of their medical records. Rigorous methodological approaches however were employed leading to the results that were generated in this novel work of comparing PCC utilization and pregnancy outcomes in a teaching hospital in Ghana Future studies involving large numbers could curtail some of these limitations.

## Data Availability

The raw data supporting the conclusions of this article will be made available by the authors, without undue reservation.
